# Isolated Renal Mucormycosis Masquerading as Bacterial Pyelonephritis in a Diabetic Patient: A Case Report

**DOI:** 10.7759/cureus.96487

**Published:** 2025-11-10

**Authors:** Yeshwanth Mohan Yelavarthy, Mukta Wyawahare, Pampa C Toi, Sreerag Sreenivasan Kodakkattil, Nirmal Raj Rajaram

**Affiliations:** 1 General Medicine, Jawaharlal Institute of Postgraduate Medical Education and Research, Puducherry, IND; 2 Pathology, Jawaharlal Institute of Postgraduate Medical Education and Research, Puducherry, IND; 3 Urology and Renal Transplantation, Jawaharlal Institute of Postgraduate Medical Education and Research, Puducherry, IND

**Keywords:** amphotericin b, bacterial pyelonephritis, diabetes mellitus, nephrectomy, renal mucormycosis

## Abstract

Isolated renal mucormycosis is a rare, aggressive fungal infection primarily affecting immunocompromised individuals. It often presents diagnostic challenges, mimicking bacterial infections like pyelonephritis. We report the case of a 45-year-old diabetic man presenting with flank pain and fever. Imaging suggested emphysematous pyelonephritis grade 3B. Initial treatment with broad-spectrum antibiotics failed, and blood and urine cultures remained sterile. Nephrectomy revealed angioinvasive mucormycosis. Despite antifungal therapy and nephrectomy, the patient succumbed to the infection. Our case highlights that renal mucormycosis can mimic bacterial pyelonephritis, delaying diagnosis. Clinicians should consider mucormycosis in diabetic patients with persistent symptoms and sterile cultures. Early surgical intervention and antifungal therapy are the mainstay.

## Introduction

Mucormycosis is a rare fungal infection often seen in immunocompromised hosts. The primary disease begins in the upper or lower airways and is usually classified as an airborne infection [1]. While the rhinocerebral and pulmonary forms of the disease have been widely documented, the occurrence of isolated renal mucormycosis is relatively uncommon, posing unique diagnostic and therapeutic challenges.

## Case presentation

**Figure 1:**
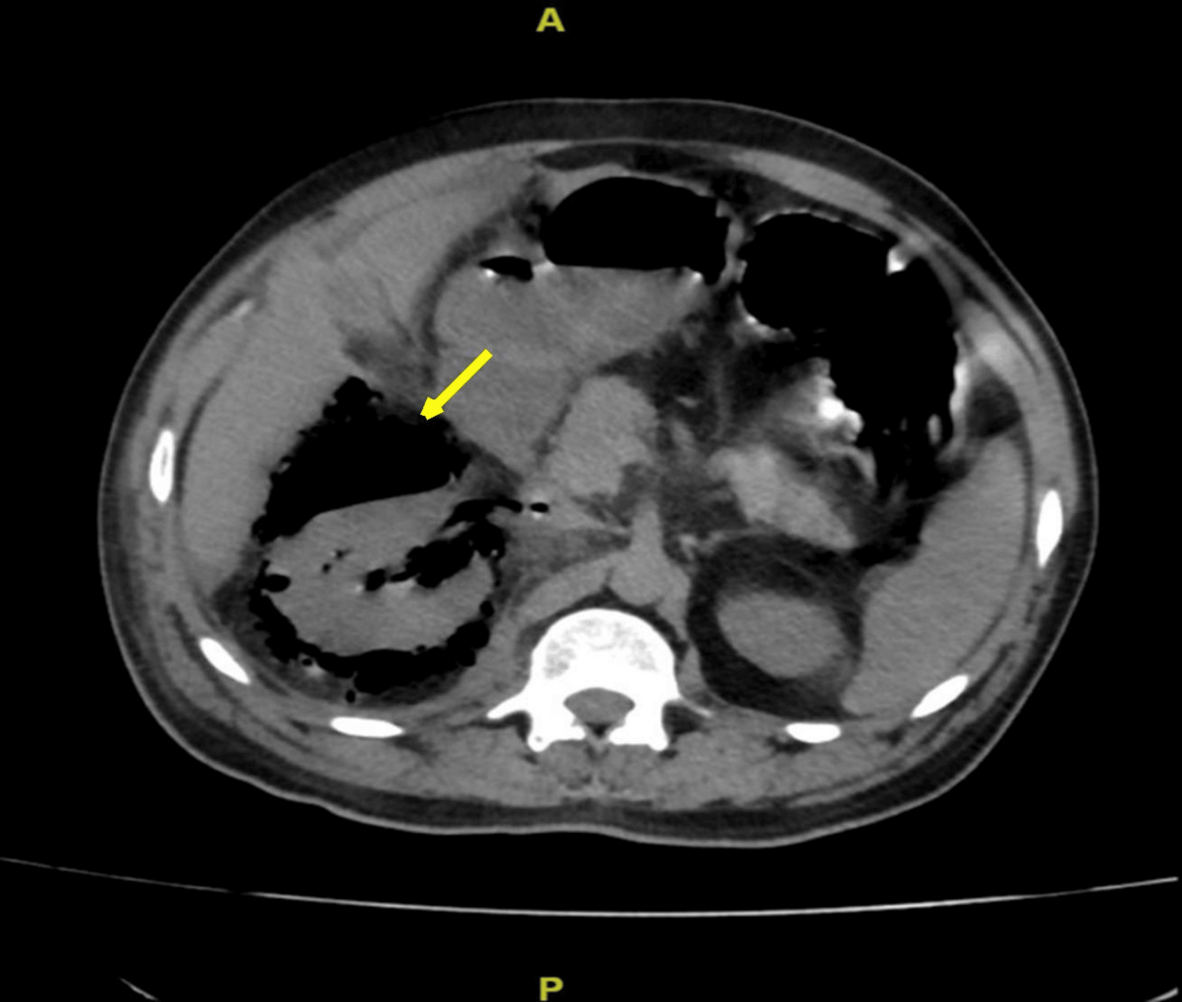
Computed tomography image (axial section) of the abdomen showing extensive gas within the right renal parenchyma and perinephric space and extending into the pararenal space, consistent with emphysematous pyelonephritis grade 3B

**Figure 2:**
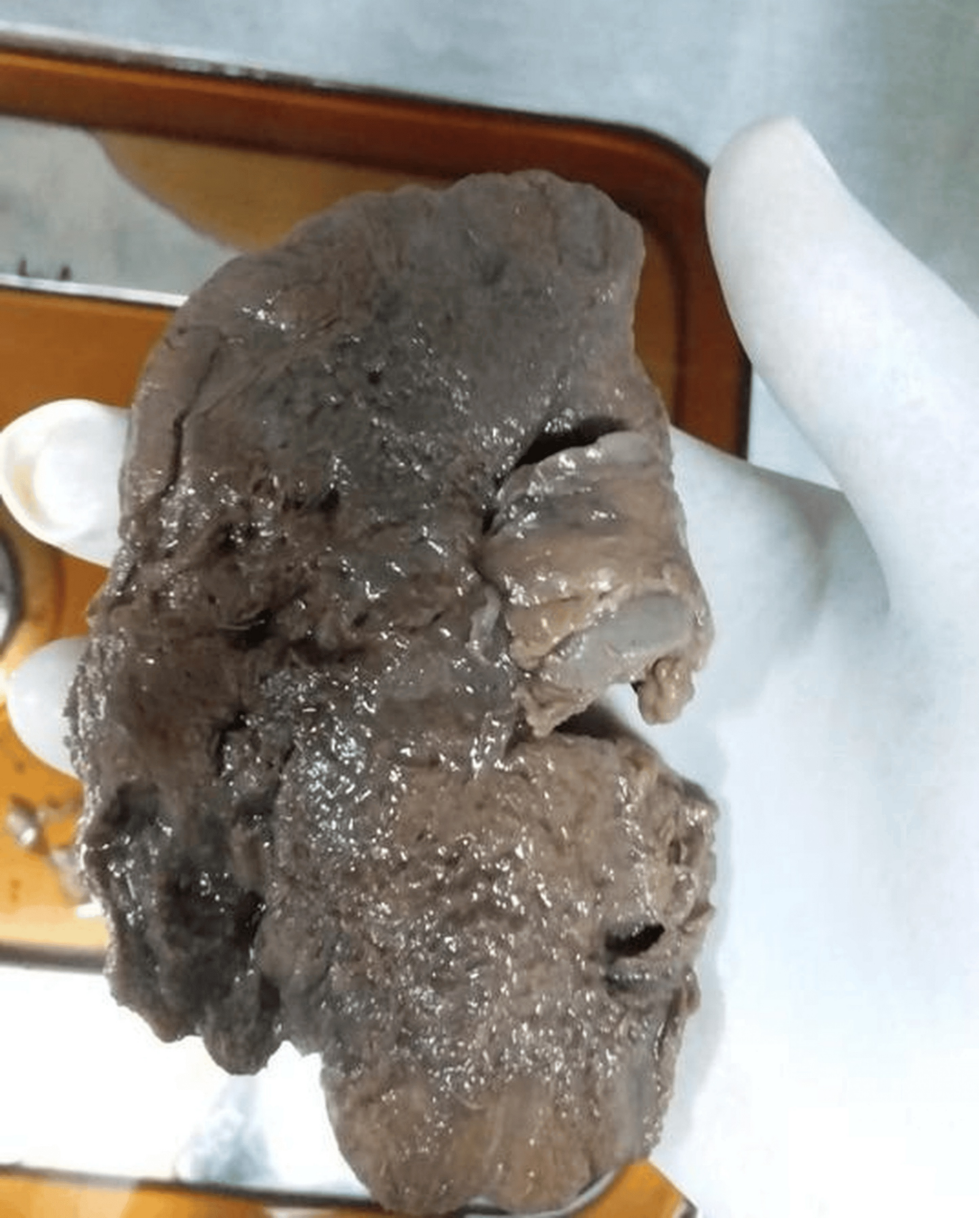
Right nephrectomy specimen showing a markedly enlarged and distorted kidney with extensive areas of necrosis and loss of normal renal architecture

**Figure 3:**
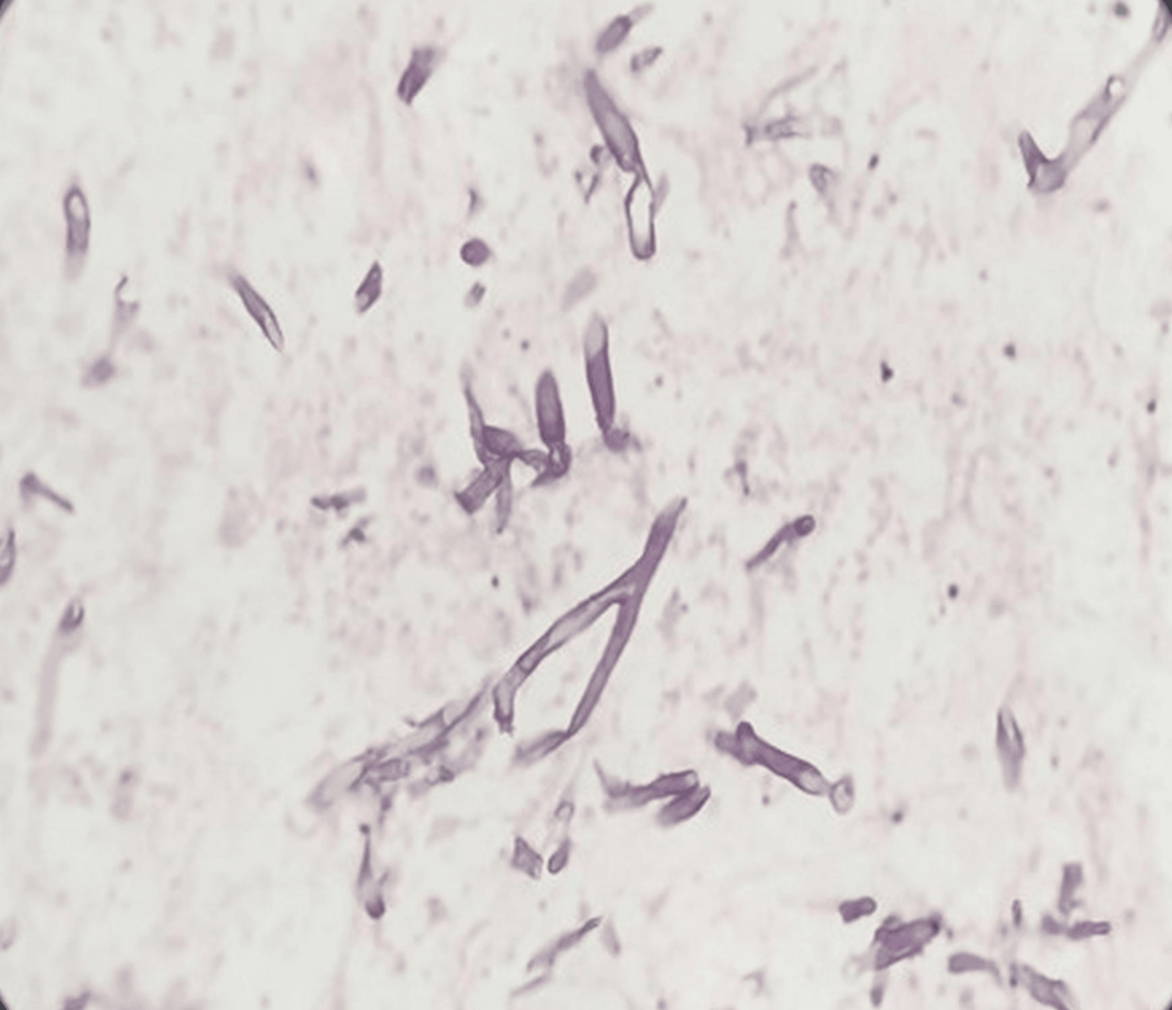
Histopathological image (H&E stain, 200×) showing broad, aseptate, right-angle branching fungal hyphae consistent with mucormycosis infiltrating the necrotic renal parenchyma

We present a 45-year-old diabetic man who presented with right-sided flank pain and fever for three days. He had a four-year history of type 2 diabetes, managed with oral hypoglycemic drugs, with persistently poor glycemic control. At admission, he had tachycardia (heart rate of 132 bpm), and his temperature was 104℉. On examination, he had right renal angle tenderness. Laboratory investigations revealed hemoglobin of 12.1 g/dL, leukocytosis (21,400 cells/μL) with neutrophilia (88%), and elevated random blood sugar (452 mg/dL), HbA1c (13%), serum urea (190 mg/dL), and creatinine (3.2 mg/dL). Furthermore, serum electrolytes and liver function tests were normal except for low albumin levels (3 g/dL). Both CRP and procalcitonin were elevated, 70 mg/L and 20.4 ng/mL, respectively. Urinalysis and microscopy revealed 1+ proteinuria, 5-10 red blood cells per high-power field (RBCs/hpf), and plenty of pus cells. Both urine and blood cultures were subsequently reported as sterile. Ultrasound and computed tomography (CT) of the abdomen (Figure 1) were done, which were suggestive of right emphysematous pyelonephritis grade 3B [2]. He was empirically started on intravenous amikacin and piperacillin-tazobactam as per our institute policy, and, in view of uremia and severe metabolic acidosis, hemodialysis was initiated. Under ultrasound guidance, a percutaneous drain was placed in the right kidney. As his condition gradually deteriorated, requiring pressor support and mechanical ventilation, a right nephrectomy was subsequently performed (Figure 2). Histopathological examination revealed angioinvasive mucormycosis (Figure 3). Following this, seven days after the initial presentation, he was started on liposomal intravenous amphotericin B at 10 mg/kg/day. Despite these measures, he ultimately succumbed to the infection.

## Discussion

Mucormycosis has garnered increasing attention in the medical community due to its rapidly progressive nature and high mortality rates. It is primarily caused by a group of fungi known as *Mucorales*, with *Rhizopus* species being the most prevalent. The disease typically affects individuals with underlying immunocompromising conditions, such as uncontrolled diabetes mellitus, organ transplantation, or hematological malignancies [1]. The increased susceptibility to mucormycosis in individuals with uncontrolled diabetes mellitus is attributed to the dysfunctional neutrophil activity and compromised phagocytic capabilities associated with hyperglycemia, which collectively diminish the host's defense against fungal pathogens [3]. This predisposition is further exacerbated by the angioinvasive nature of *Rhizopus*, which can lead to thrombosis and infarction of renal tissue, thereby compounding the organ damage.

The rhinocerebral and rhinomaxillary forms of the disease are the most commonly reported, affecting the nasal and paranasal sinuses, as well as the oral and palatal regions [4]. However, isolated renal involvement is a rare manifestation, accounting for only a small percentage of all mucormycosis cases. A review of the existing literature reveals several case reports and case series documenting instances of isolated renal mucormycosis [5,6].

Early diagnosis is crucial, as the infection can rapidly progress, leading to renal infarction, abscess formation, and, eventually, disseminated disease [7]. Of note, both blood and urine cultures remained sterile throughout the initial evaluation, which delayed the consideration of a fungal etiology in the index case. Surgical debridement and prompt antifungal therapy are the cornerstones of treatment for isolated renal mucormycosis. Surgical management typically involves nephrectomy, which may be necessary to control the infection and prevent further spread. However, data on the outcomes following nephrectomy for isolated renal mucormycosis remain sparse.

## Conclusions

Mucormycosis may masquerade as bacterial pyelonephritis. Clinicians should suspect mucormycosis when a patient is not improving with broad-spectrum antibiotics, and it should be managed aggressively with surgical debridement and antifungal medications.

## References

[1] Sharma A, Goel A (2022). Mucormycosis: risk factors, diagnosis, treatments, and challenges during COVID-19 pandemic. Folia Microbiol (Praha).

[2] Huang JJ, Tseng CC (2000). Emphysematous pyelonephritis: clinicoradiological classification, management, prognosis, and pathogenesis. Arch Intern Med.

[3] Fernandez JF, Maselli DJ, Simpson T, Restrepo MI (2013). Pulmonary mucormycosis: what is the best strategy for therapy?. Respir Care.

[4] Verma M, Sharma R, Verma N, Verma K (2020). Rhinomaxillary mucormycosis presenting as palatal ulcer: a case report with comprehensive pathophysiology. J Oral Maxillofac Pathol.

[5] Bhadauria D, Etta P, Chelappan A (2018). Isolated bilateral renal mucormycosis in apparently immunocompetent patients-a case series from India and review of the literature. Clin Kidney J.

[6] Saran S, Naranje K, Gurjar M, Bhadauria D, Kaul A, Poddar B (2017). Isolated renal mucormycosis in immunocompetent children: a report of two cases. Indian J Crit Care Med.

[7] Sharma R, Shivanand G, Kumar R, Prem S, Kandpal H, Das CJ, Sharma MC (2006). Isolated renal mucormycosis: an unusual cause of acute renal infarction in a boy with aplastic anaemia. Br J Radiol.

